# Aperiodic Linear Networked Control Considering Variable Channel Delays: Application to Robots Coordination

**DOI:** 10.3390/s150612454

**Published:** 2015-05-27

**Authors:** Carlos Santos, Felipe Espinosa, Enrique Santiso, Manuel Mazo

**Affiliations:** 1 Electronics Department, Polytechnics School, University of Alcala, Campus Universitario, Ctra. Madrid-Barcelona, Km. 33,600, 28871. Alcalá de Henares, Madrid, Spain; E-Mails: espinosa@depeca.uah.es (F.E.); santiso@depeca.uah.es (E.S.); 2 Delft Center of Systems and Control, Delft University of Technology, Mekelweg 2, 2628 CD Delft, The Netherlands; E-Mail: M.Mazo@tudelft.nl

**Keywords:** aperiodic control implementation, adaptive self-triggered control, wireless cyber-physical systems, variable channel delays, networked robots

## Abstract

One of the main challenges in wireless cyber-physical systems is to reduce the load of the communication channel while preserving the control performance. In this way, communication resources are liberated for other applications sharing the channel bandwidth. The main contribution of this work is the design of a remote control solution based on an aperiodic and adaptive triggering mechanism considering the current network delay of multiple robotics units. Working with the actual network delay instead of the maximum one leads to abandoning this conservative assumption, since the triggering condition is fixed depending on the current state of the network. This way, the controller manages the usage of the wireless channel in order to reduce the channel delay and to improve the availability of the communication resources. The communication standard under study is the widespread IEEE 802.11g, whose channel delay is clearly uncertain. First, the adaptive self-triggered control is validated through the TrueTime simulation tool configured for the mentioned WiFi standard. Implementation results applying the aperiodic linear control laws on four P3-DX robots are also included. Both of them demonstrate the advantage of this solution in terms of network accessing and control performance with respect to periodic and non-adaptive self-triggered alternatives.

## 1. Introduction and Motivation

A cyber-physical system (CPS) is an orchestration of physical systems, computers and network capabilities [1]. Network control systems (NCS) are an important class of CPS, in which physical processes are controlled using networked actuators, sensors and controllers [2–8]. The performance of these systems depends not only on the designed control algorithm, but also on the scheduling of resources in the shared network [8]. This has motivated an increasing interest in optimizing the usage of shared resources while maintaining the desired control performance in NCS [7–15]. This paper proposes a novel aperiodic linear control algorithm for NCS adapted to the variable communication channel delays.

With the advent of NCS, greater functionality is expected, and control loops no longer have at their disposal dedicated computational and communication resources [2,9,16]. While traditionally, the implementation aspects were ignored at the design state, this can no longer be done. New techniques abandoning the periodic paradigm in favor of strategies based on sampling only when necessary have been proposed to achieve a better use of shared resources [9,16–22]. Two of the most important techniques are event-triggered and self-triggered control (STC). In event-triggered control implementations [17,19,20], the current state of the plant is measured constantly in order to decide when control updates must be triggered. On the other hand, self-triggered control does not require the continuous monitoring of the state of the plant. Self-triggered control emulates event-triggered implementations, predicting when the system needs to be updated [16,18]. In this way, the controller determines the next update time from the last measurement. In this work, STC is the aperiodic alternative chosen by the authors.

Up to date, previous event-triggered or self-triggered proposals dealing with variable delays used an upper bound of the delay (maximum value) to guarantee stability [10,11,13]. This is a clear conservative solution, as the maximum delays often occur only when the network channel is congested. In [11], three different aperiodic control algorithms are presented over the IEEE 802.15.4 standard. These algorithms combine event-triggered and self-triggered strategies. In [10], a model-based network control system and event-triggered control are combined under a single framework. In [13], an event-based controller is used to reduce the network bandwidth usage considering the effect of random time-varying network-induced delays. In contrast to previous works, in this paper, the triggering condition is adapted to the actual network occupancy. Consequently, the triggering condition is chosen according to the measured network delay, allowing one to accommodate the number of control executions to the channel load.

Among CPS, remote control applications [12] and cooperative guidance of mobile units [23] are especially interesting. Examples of remote control can be found in several articles in the literature. In [7], a hybrid distributed topology control scheme for vehicular sensor networks is presented. A cooperative motion and task planning of mobile robots under local linear temporal logic specifications is given in [14]. In [20], a distributed control algorithm based on event-triggered communications is designed and implemented to bring a set of mobile robots into different formations. A robotic vehicle is bilaterally tele-operated by means of a power-based passivity controller with periodic sampling in [24]. In [5], a network is comprised of a swarm of wirelessly-connected mobile robots equipped with various sensors.

In the context of robotics cooperation and tele-operation, it is of foremost importance to minimize the computational load of the on-board electronics system [15,25,26], as well as to reduce the communications burden. In a previous work [27], a conservative design dealing with the maximum delay was evaluated and implemented. That solution achieves a significant reduction in the network traffic when only a robot is remotely controlled. However, because of the conservative flavor, the higher the number of robotic units sharing the WiFi channel, the worse the performance due to the increasing maximum delay. The challenge of optimizing the shared resources in a multi-robotic remote control application justifies the objective of this paper. For that, in this work, the authors propose the adaptation of the aperiodic triggering mechanism to the actual state of the channel load. As a proof of concept, four Pioneer P3-DX robots are remotely controlled by the same computer through the widespread IEEE 802.11g standard. This is a linear control approach that allows focusing the paper on the management of the channel usage and the compensation of current delays through an adaptive self-triggered proposal. The remote center (RC) runs a self-triggered velocity servo-controller for each unit. The non-conservative approach relaxes the performance requirements of the controller when the network is congested.

The adaptive triggered mechanism involves two important parameters: a local parameter, which is the deviation of the robot state vector from its equilibrium point (|*x*(*t*) − *x_eq_*|) (fully described in [12]), and a global parameter, which is the actual network communication delay (*τ*). In this way, the wireless network traffic is greatly reduced, while the tracking performance is not significantly degraded.

The rest of the paper is organized as follows: Section 2 presents a review of self-triggered control for linear time-invariant systems. Section 3 details the adaptive self-triggered condition to deal with the variable network delays. Section 4 describes the experimental setup remotely controlling four robots. Simulation and experimental results are shown in Section 5. Finally, Section 6 summarizes the contribution of the paper and gives a hint about future developments.

## 2. Mathematical Background

This section reviews the notions of self-triggered control for linear time-invariant linear systems on the continuous time domain. The specific problem statement is introduced, and the interest of applying an adaptive triggering condition to solve it is discussed.

### 2.1. Preliminaries

Denote by ℝ^+^ the positive real numbers being ${\text{R}}_{0}^{+}={\text{R}}^{+}\cup \left\{0\right\}$ and by ℕ the natural numbers. The usual Euclidean (*l*_2_) vector norm is represented by | · |.

A classic result on stability theory for linear systems is reminded in the following theorem.

#### Theorem 1.

*A linear system ẋ(t;* = *Ax*(*t*), *x*(*t*) ∈ ℝ*^n^*, *A* ∈ ℝ*^n^*^×^*^n^*; *is globally exponentially stable (GES), i.e.*, ∃ *M*, λ ∈ ℝ^+^, *such that* |*x*(*t*)| ≤ *Me*^−λ^*^t^*|*x*(0)|, *if and only if there exist positive definite matrices P, Q* ∈ ℝ*^n^*^×^*^n^, such that:*

(1) $$A^{T}P+PA\leq -Q$$

*Then, the function V (t) = x(t)^T^ Px(t) is said to be a Lyapunov function for the system*.

### 2.2. Self-Triggered Control Keys

The essential aspects of self-triggered control for linear time-invariant systems are reviewed in this subsection. This helps the reader to better understand the current proposal; a more detailed description about this aperiodic control strategy can be found in [16].

A linear time-invariant system is modeled by:

(2) $$\begin{array}{c} \dot{x}\left(t\right)=Ax\left(t\right)+Bu\left(t\right)\\ y\left(t\right)=C_{x}\left(t\right) \end{array}$$

where *A* ∈ ℝ^n×n^, *B* ∈ ℝ*^n^*^×^*^r^*, *C* ∈ ℝ*^m^*^×^*^n^* are the characteristic matrices and *x*(*t*) ∈ ℝ*^n^, u*(*t*) ∈ ℝ*^r^* and *y*(*t*) ∈ ℝ*^m^* are the state, input and output vectors, respectively. If the pair (*A, B*) is stabilizable, a linear feedback controller *u*(*t*) = *Kx*(*t*) rendering the closed loop GES can be found; hence, according to Theorem 1, there exists a Lyapunov function of the form:

(3) $$V\left(t\right)=x^{T}\left(t\right)Px\left(t\right)$$

where *P* is a positive define matrix satisfying the Equation (1) for a positive definite matrix *Q* chosen by the designer.

However, in digital implementations, the input *u* is available only at discrete times *t_k_*. That is, at times *t_k_*, the controller is recomputed with fresh measurements, and the plant input is kept constant (ZOH) until a new measurement is received, *i.e.*,

(4) $$\begin{array}{cc} u\left(t\right)=Kx\left(t_{k}\right), & t\in \left[t_{k},t_{k+1}\right] \end{array}$$

The objective of the self-triggered control strategy is to minimize the number of updates preserving the closed loop system stability The sequence of update times *t_k_* is implicitly defined as the times at which some triggering condition is violated.

To guarantee stability, a performance function $S:{\text{R}}_{0}^{+}\times {\text{R}}^{n}\to {\text{R}}_{0}^{+}$ upper-bounding the evolution of *V* is used. This way, the update times *t_k_* are determined by the time instants at which:

(5) $$\begin{array}{cc} V\left(t,x_{t_{0}}\right)\leq S\left(t,x_{t_{0}}\right), & t\geq t_{0} \end{array}$$

is violated.

Provided that Equation (5) holds and *S* is decaying over time, the closed loop system is stabilized, with a decay rate of its Lyapunov function *V* no lower than the specified through *S*. The use of a function *S* is suggested, given by:

(6) $$S\left(t\right):=x_{s}{\left(t\right)}^{T}Px_{s}\left(t\right)$$

(7) $${\dot{x}}_{s}\left(t\right)=\begin{array}{cc} A_{s}x_{s}\left(t_{k}\right), & t\in \end{array}\left[t_{k},t_{k+1}\right]$$

(8) $$x_{s}\left(t_{k}\right)=x\left(t_{k}\right)$$

where *A_s_* is a Hurwitz matrix satisfying the Lyapunov equation:

(9) $$A_{s}^{T}P+PA_{s}=-R$$

where **R** is a definite positive matrix.

The inter-execution times (*t_k_*_+1_ − *t_k_*) must be lower bounded by some positive quantity *t_min_* [16], avoiding the Zeno executions [28] of the hybrid system. In order to guarantee inter-execution times greater than zero, it is sufficient to design *R* < *Q*, which guarantees *V̇*(*t_k_*) < *Ṡ*(*t_k_*).

As is described in [18], knowing the dynamics of the system and the measurement at time *t_k_*, the evolution of the state *x*(*t_k_* + *t*), *t* ∈ ℝ^+^ can be predicted. Owing to the digital implementation of the controller, the triggering condition (5) is checked with a discretization step (Δ). Thus, one can compute ahead of time *V* and *S* at times separated Δ units and check if:

(10) $$\begin{array}{cc} V\left(t_{k}+t_{\min}+p\Delta ,x_{t_{k}}\right)\leq S\left(t_{k}+t_{\min}+p\Delta ,x_{t_{k}}\right), & p\in \left[0,1,\ldots ,N\right] \end{array}$$

for *N* ∈ ℕ some pre-specified horizon. Then, one can compute *t_k_*_+1_ = *t_k_* + *t_min_* + *p*Δ, such that: either *p* = *N* or:

(11) $$V\left(t_{k}+t_{\min}+\left(p+1\right)\Delta ,x_{t_{k}}\right)>S\left(t_{k}+t_{\min}+\left(p+1\right)\Delta ,x_{t_{k}}\right)$$

Note that the discretization time Δ presents a trade-off between the computational complexity of the implementation and the performance; for detailed information see [16].

Considering the application of remote control of multiple robots, each one has to track piecewise constant reference signals (*y_ref_*). This means that for non-zero references, the equilibrium of the system is:

(12) $$x_{eq}=-{\left(A+BK\right)}^{-1}By_{ref}$$

This poses no problem, as the new equilibrium point is easily computed by the following change of coordinates: *x̃* = *x* − *x_eq_*.

## 3. Self-Triggered Condition Adapted to Network Load

In the first place, a strategy to choose a proper *S* function is presented. To ensure that the condition 0 < *R* < *Q* holds:

(13) $$\begin{array}{cc} R=\sigma Q, & 0<\sigma <1 \end{array}$$

Thus, according to Equation (9), the decay rate of the Lyapunov function Sdepends on the sigma value.

The choice of σ provides a trade-off between the number of updates and the stability requirements. In a qualitative way, it can be said that for *σ →* 0, significant reduction of controller updates will be achieved, as well as corresponding performance degradation. On the other hand, for σ → 1, one obtains a better performance at the cost of an increase in the number of updates.

The main idea behind the adaptive triggering condition is to take advantage of the benefits of the reduction of transmissions without losing performance. Consequently, the value of σ is selected depending on the current network delay *τ* and the deviation of the state vector from its equilibrium point (|*x*(*t*) − *x_eq_*|).

When *τ* is greater than the average delay, the system works with the lowest range of the triggering condition (σ → 0) to reduce the transmissions and thus attenuate the channel congestion. On the other hand, when *τ* is smaller than the average delay, the adaptive controller changes to the highest range (σ → 1) to achieve a fast response of the feedback control system. Moreover, the value of σ is also adjusted according to the tracking error [12]. During the transient response of the system, σ is set to a value larger than its value at steady state. Therefore, the system obtains a fast response when it is far from its equilibrium point and a reduction in the controller updates when it is close to it. The threshold values delimiting the mentioned ranges of *τ* and |*x*(*t*) − *x_eq_*| are selected by the designer depending on the specific performance requirements and the available resources.

### 3.1. Communication Procedure

In remotely-controlled systems, data transfer from the sensors to the controller (remote center) and then to the actuator (plant) is generally characterized by time-varying delays [4].

In order to define threshold values delimiting the network delay ranges, the authors model these network delays by a gamma probability distribution [29], because a gamma distribution model for time delays in the network fits relatively well with real indoor environments. The following parameters are used to model the network delay:

(1) The current delay *τ* is measured in each bidirectional communication between the robot and the remote center.
(2) The minimum delay *τ_MIN_* is the lowest value of the network delay.
(3) The maximum delay *τ_MAX_* is the highest value of the network delay. It is the worst scenario to guarantee the system stability.

The proposed strategy is an extension of the one described in [27] dealing with variable delays. Basically, to compensate the channel delay, it is required to transmit a measurement at the time:

(14) $$t_{s_{k}}=t_{k}-\hat{\tau }$$

being *τ̂* the time that guarantees the signal reception before *t_k_*. Thus, the remote controller receives the measurements early enough to compute the control signal *u*(*t_k_*), assuring that the plant is going to receive the new control input before *t_k_*. Consequently, this approach has a slight predictive flavor and requires the remote controller to estimate the value of the state vector at the time *t_k_* based on the measurement sent by the plant at *t_sk_*:

(15) $$A_{\hat{\tau }}=e^{A\hat{\tau }}$$

(16) $$B_{\hat{\tau }}=\int_{0}^{\hat{\tau }}e^{A\left(\hat{\tau }-\lambda \right)}Bd\lambda$$

(17) $$\hat{x}\left(t_{s_{k}}+\hat{\tau }\right)=A_{\hat{\tau }}x\left(t_{s_{k}}\right)+B_{\hat{\tau }}u\left(t_{k-1}\right)$$

where *x̂*(*t_k_*) is the predicted value of *x*(*t_k_*).

Working over the maximum delay as in [27] leads to a clear conservative solution, because it is the worst case scenario. In this case, the *τ_MAX_* is restricting the performance due to the *t_min_* value obtained through the performance function *S*(*t*) having to be greater than this delay in order to guarantee stability.

**Figure 1:**
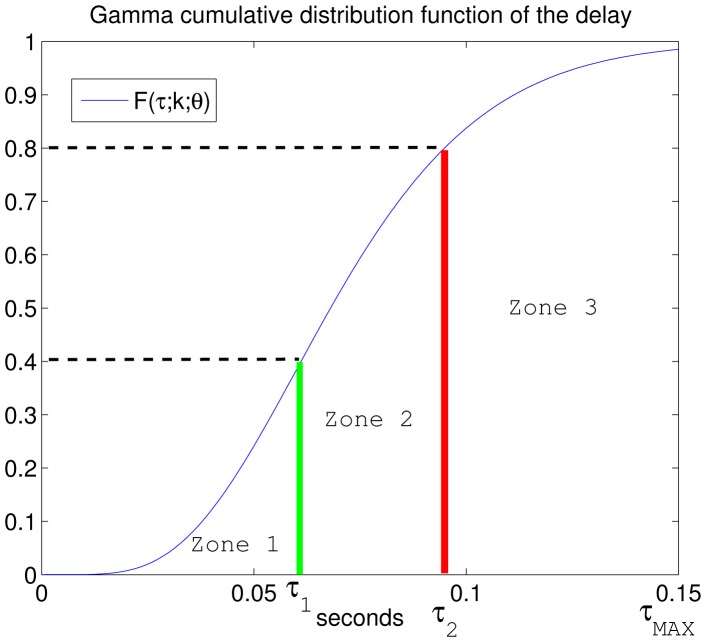
Gamma cumulative distribution function of the channel delay. Case study: *L* = 3.

Owing to the fact that the actual network delay is not measured until the robot receives the control information, the possible delays are classified into zones and compensated for the worst case scenario of each zone, thus guaranteeing stability. Therefore, the network delay range is divided into *L* zones with a single σ value associated with each one. The definition of the different zones is supported by the gamma cumulative distribution function. The election of *L* presents a trade-off between the computational complexity of the control law implementation and the flexibility to take advantage of the actual channel status. Nonetheless, the number of *L* zones increases *L* times the amount of data sent over the network. In the case of packet-based networks, it is not a problem if the size of one packet is not exceed [3]. If the network is non-packet-based, the designer should be aware of the drawback regarding the increase of the data amount. An example where *L* = 3 is shown in Figure 1.

The current network delay *τ* cannot be estimated before the remotely-controlled system sends and receives the information. This forces the remote center to calculate a control signal for each different range. All of them are sent to the plant, but once it receives the control information, it measures the actual delay and decides which control action must be applied.

The adaptive control strategy determines *L* update times as:

(18) $$\begin{array}{cc} t_{i,k}=t_{k}+h\left(\sigma_{i}\right), & i\in \left[1,2,\ldots ,L\right] \end{array}$$

where the times *h*(*σ_i_*) are evaluated by the instants at which the condition:

(19) $$\begin{array}{cc} V\left(t,x_{t_{0}}\right)\leq S\left(t,x_{t_{0}},\sigma_{i}\right), & t\geq t_{0} \end{array}$$

is violated.

As was explained, the parameter *σ_i_* is dependent on *τ* and |*x*(*t*) − *x_eq_*|, and *σ_i_* ∈ [0, 1] guarantees:

(20) $$\begin{array}{cc} h\left(\sigma_{i}\right)>0, & \forall t \end{array}$$

**Figure 2:**
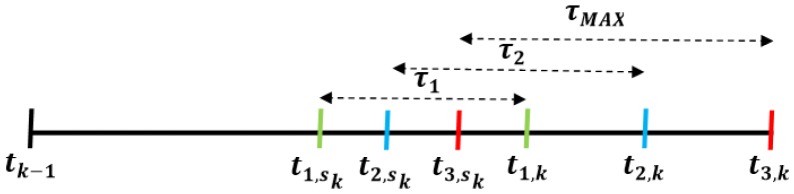
Possible transmission instant *t_i,sk_.* Case study: *L* = 3.

To prevent the delay effect, the plant sends its measurement vector at the time *t_sk_* taking into account the *L* possible values of *t_k_* (as shown in Figure 2):

(21) $$t_{s_{k}}=\min\left(t_{1,k}-\tau_{1},\ldots ,t_{i,k}-\tau_{i},\ldots ,t_{L,k}-\tau_{MAX}\right)$$

where *τ_i_* for *i* ∈ [1, 2,.., *L*] are the greater delay values of the *L* zones.

**Figure 3:**
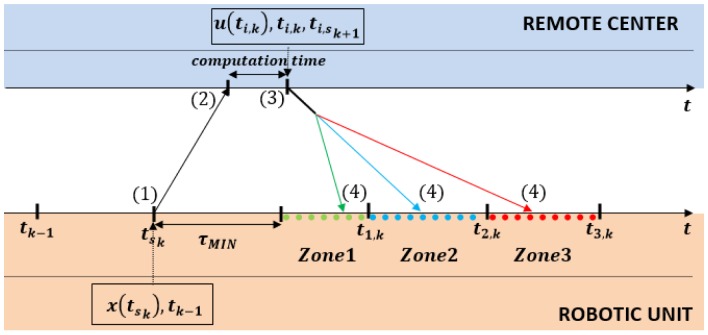
Control communication protocol (example for *L* = 3).

Figure 3 depicts a detailed sketch of the communication protocol between a controlled system (robotic unit) and the remote controller. The protocol consists of four steps:

Step 1: At time *t_sk_* the robotic unit sends its measurements *x*(*t_sk_*) and the last update time *t_k_*−1 to the remote controller.
Step 2: The remote controller receives the *x*(*t_s_k__*) vector and predicts all possible values of *x̂*(*t_i,k_*) according to Equation (17). For all possible *x̂*(*t_i,k_*) values, the control signals *u*(*t_i,k_*) are computed, and the self-triggered scheduler calculates the next sampling times *t_i,k_*_+1_ for each one of the *L* possible zones. Finally, the controller sends through the wireless channel the following information: *t_i,k_*, *u*(*t_i_*_,_*_k_*) and *t_i_*_,_*_sk_*_+1_.
Step 3: The robotic unit receives the information with the current delay *τ*. The correct zone (from *L*) is identified by observing the actual network delay, and only the proper control action *u*(*t_k_*) and time *t_sk_*_+1_ are considered. Then, the system waits until the corresponding time *t_k_*.
Step 4: The robot applies the proper control signal *u*(*t_k_*) at the precise time *t_k_*.

#### Remark 1

*It is noteworthy that synchronization is not necessary between the remote controller and each robot to measure the total network delay, because this delay is measured from the time a robot sends its measurements (Step 1) until it receives itself the control information (Step 3)*.

### 3.2. Selection of the σ Values

There exists a clear relation between the channel delay (*τ*) and the σ values. The delay constrains the performance due to the *t_min_* value, which is obtained through the performance function and must be greater than the delay in order to guarantee stability. In turn, according to Theorem 5.1 [16] and considering Equation (5) of this paper, it can be observed that the *t_min_* depends on a. The greater the σ, the lower the *t_min_*. To ease the understanding of the relation between *τ* and σ, the limit σ values should be taken into account:

#### Remark 2.

*If σ* = 0, *the system would be bounded in a Lyapunov set, guaranteeing V*(*x*(*t_k_*)) = *V*(*x*(*t_k_*_+1_). *Using this strategy, the asymptotic stability is not assured, and it can only be said that the system will not be unstable. With this σ value, the maximum t_min_is obtained, and it is equal to the maximum channel delay* (*τ_MAX_*) *that the network control system can compensate with this control strategy*.

#### Remark 3.

*If σ* = 1, *the system would be forced to update the control signal continuously. It is the equivalent to the continuous control approach; this* σ *value is impossible to implement in a digital platform due to the time between updates being null.*

**Figure 4:**
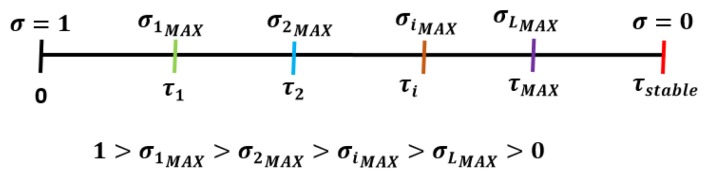
Relation between the network delay and the σ values.

Figure 4 depicts the key aspects to select the σ value according to the current network delay. The first step checked by the designer is to find the maximum allowable delay (*τ_stable_*); all of the delays have to be smaller than this one, assuring stability Next, the number of delay zones are fixed, and with them, the corresponding partial limit delay *τ_i_* is known. Then, the maximum σ value concerning each zone is calculated. Finally, the designer chooses a σ value taking into account that it must be equal to or lower than the maximum one previously calculated. For example: σ*_i_* ∈]0, σ*_iMAX_*] where *t_min_*(σ*_iMAX_*) = *τ_i_*.

## 4. Experimental Setup

To demonstrate the proposal's benefits in a telerobotic context, the described strategy is applied on four P3-DX mobile robots linked to the remote center by the same WiFi channel (IEEE 802.11g).

### 4.1. Plant Model and Servo Controller Design

The delay is not included as part of the plant model. That is, the P3-DX robot is locally modeled, without wireless channel effects. Each robotic unit incorporates the lowest control level associated with the active wheels and a digital observer to recover the full state of the robot from odometric information providing filtered linear and angular velocities. The plant model is obtained with standard system identification techniques [30] and validated through experimental trials. Linear and angular velocity references (components of input vector *u*(*t*)) are sent to the robot, and the open-loop robotic response (linear and angular velocities as output vector *y*(*t*)) is registered. From experimental data, the following continuous state-space model of the P3-DX robotic unit is obtained.

(22) $${\dot{x}}_{r}\left(t\right)=Ax_{r}\left(t\right)+Bu\left(t\right)=\left[\begin{array}{ll} -4.094 & -0.015\\ -0.008 & -5.042 \end{array}\right]x_{r}\left(t\right)+\left[\begin{array}{ll} -4.159 & -0.002\\ -0.001 & -5.057 \end{array}\right]u\left(t\right)$$

(23) $$y\left(t\right)=Cx_{r}\left(t\right)=\left[\begin{array}{ll} 1 & 0\\ 0 & 1 \end{array}\right]x_{r}\left(t\right)$$

where:

- *x_r_*(*t*) ∈ ℝ^2^ is the plant state vector (current linear and angular velocities of the robot);
- *u*(*t*) ∈ ℝ^2^ is the input state vector (velocity command sent to the robot);
- *y*(*t*) ∈ ℝ^2^ is the filtered measurement vector obtained from the observer (matching the state vector).

A servo system is designed to properly track the gradual variation of linear and angular references for each robot. The LQRdesign technique [31] is applied to each robot. The robot dynamics (*x_r_*) extended with the integrator dynamics (*x_n_*) allows one to determine *K_I_* and *K_R_* gain matrices according to:

(24) $$\left[\begin{array}{c} {\dot{x}}_{r}\left(t\right)\\ {\dot{x}}_{n}\left(t\right) \end{array}\right]=\left[\begin{array}{ll} A & 0\\ -C & 0 \end{array}\right]\left[\begin{array}{l} x_{r}\left(t\right)\\ x_{n}\left(t\right) \end{array}\right]+\left[\begin{array}{l} B\\ 0 \end{array}\right]\left[\begin{array}{cc} K_{R} & K_{I} \end{array}\right]\left[\begin{array}{l} \begin{array}{l} x_{r}\left(t\right)\\ x_{n}\left(t\right) \end{array} \end{array}\right]+\left[\begin{array}{l} 0\\ I \end{array}\right]y_{ref}\left(t\right)$$

where:

- *x_n_*(*t*) ∈ ℝ^2^ is the integrator state vector related to the linear and angular velocities of the robot;
  The weighting matrices used in the LQR design are:
  (25) $$\begin{array}{cc} Q_{LQR}=\left[\begin{array}{ll} 0.1I_{2x2} & 0_{2x2}\\ 0_{2x2} & I_{2x2} \end{array}\right]; & R_{LQR} \end{array}=I_{2x2}$$
  and the resulting constants of the controller *K_R_* and *K_I_* are:
  (26) $$\begin{array}{cc} K_{R}=\left[\begin{array}{ll} -0.2605 & 0.0004\\ 0.0005 & -0.2234 \end{array}\right], & K_{I} \end{array}=\left[\begin{array}{cc} 1 & 0.0007\\ -0.0007 & 1 \end{array}\right]$$

The remote center, a PC sharing the wireless network with the robotic units, deals with three main tasks:

- generation of the velocities reference vector;
- calculation of the robotic control vector;
- execution of the self-triggered scheduler.

**Figure 5:**
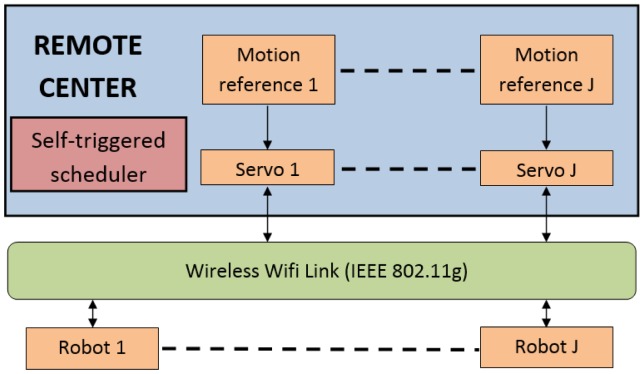
Global structure of the adaptive self-triggered strategy implemented for multiple robots that are remotely controlled.

The self-triggered scheduler is responsible for deciding when the state vector estimation has to be updated and when the control action has to be applied to each robotic unit. The higher the interval inter-executions, the lower the load of the wireless channel. Figure 5 shows the global structure of the implemented adaptive self-triggered control.

### 4.2. Communication Parameters

First, the maximum time-delay that can be compensated by the adaptive self-triggered controller is calculated. In this way, the designer has a bound on the maximum admissible delay This maximum admissible delay is 175.7 ms, which is equal to the *t_min_* obtained with σ = 0.

Different experiments are carried out to identify the gamma distribution parameters, exchanging information between the remote center and the robots. The total delay is measured from the time a robot sends its measurements to when it receives the control action. This time includes both channel delays and the remote center computation time. The maximum delay registered is 150 ms, which it is lower than the maximum admissible delay.

The next step is the selection of the gamma probability parameters that models the network delay *τ*, as can be seen in Section 3.1. The values of *τ* are classified into three zones (*L* = 3) bounded by the values of its gamma cumulative distribution function Γ(τ), as can be noticed in Figure 1: *τ_MIN_* = 0.005 *s, τ*_1_ = 0.061 *s, τ*_2_ = 0.095 *s* and *τ_MAX_* = 0.15 *s*. Then, the three ranges of network delays are discriminated by:

Zone 1: from Γ(τ*_MIN_*) = 0 to Γ(τ_1_) = 0.4.
Zone 2: from Γ(τ_1_) = 0.4 to Γ(τ_2_) = 0.8.
Zone 3: from Γ(τ_2_) = 0.8 to Γ(τ*_MAX_*) = 1.

### 4.3. Sigma Values

The designed strategy considers six different σ values combining qualitative values of network delay (three cases) and the deviation of the state vector from the equilibrium point (two different cases). The limits of the network delay ranges have been selected in the previous subsection. For the state vector drift, only low and high cases with limit value |*x*(*t*) − *x_eq_*| = 0.1 are provided. Due to the direct effect of the network delay on the shared resource (the wireless channel), it is prioritized over the deviation of the state vector from the equilibrium condition. Table 1 includes the sigma values related to the self-triggering adaptive strategy.

## 5. Results

**Figure 6:**
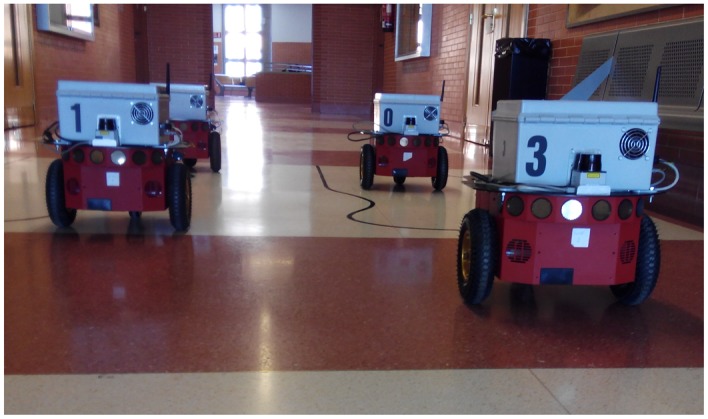
Experimental test using four P3-DX robotic units remotely controlled by a personal computer.

This section presents simulation and experimental tests using a study case of four P3-DX robotic units remotely controlled by a unique personal computer and linked through the same WiFi network (as shown in Figure 6). To evaluate the performance of the control system, the integral of the squared error (ISE) index is applied to the output tracking:

(27) $$ISE=\sum_{k=0}^{\infty }{\left|y\left(k\Delta \right)-y_{ref}\left(k\Delta \right)\right|}^{2}\Delta$$

### 5.1. Simulation Results

As the first approach, the TrueTime software tool is used to evaluate the aperiodic remote control of multiple robots considering variable channel delays [32]. TrueTime makes it possible to simulate models of communication networks and their influence on networked control loops. Different scheduling policies may be used, although in this case, the available standard in the robotics laboratory is IEEE 802.11g. The basic parameters of the experimental network are set in the TrueTime Wireless Network Block, such as: the network type, 802.11g WLAN; data rate, 4 Mbps; frame size, 808 bits; transmission power, 28 dBm; receiver signal threshold, −98 dBm.

For comparison purposes, three different implementations of the controller are considered:

(1) A periodic implementation with constant sampling period equal to the discretization step Δ = 10 ms: in this case, only the remote control of one robotic unit was simulated to be used as a reference of performance.
(3) A self-triggered implementation with two fixed triggering conditions: one close to zero (σ = 0.05) and another close to one (σ = 0.9).
(3) An adaptive self-triggered implementation applying the triggering condition described in Section 4.3.

**Figure 7:**
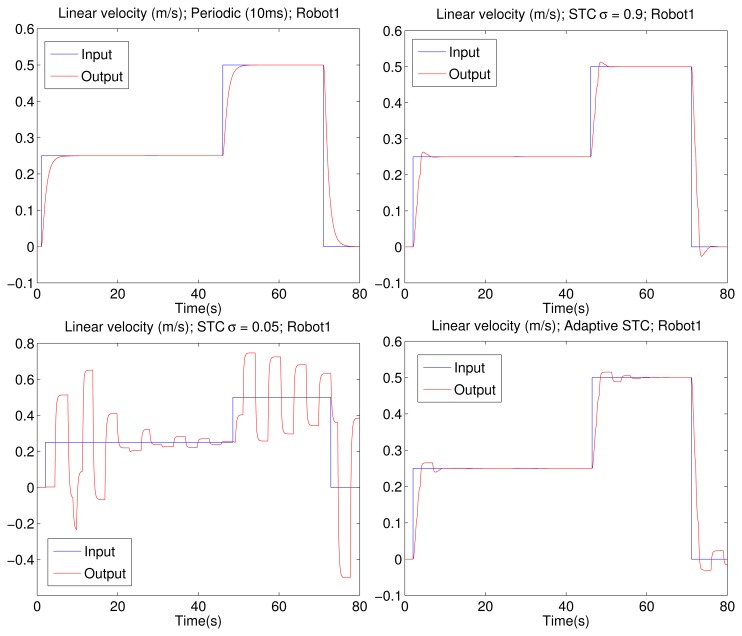
Linear velocity registered (red line) when a reference (blue line) is applied to one of the robots. Results from different implementations: periodic sampling (**Upper Left Corner**), fixed high value of the sigma parameter (**Upper Right Corner**), fixed low value of the sigma parameter (**Lower Left Corner**) and adaptive solution proposed by the authors (**Lower Right Corner**).

Figure 7 shows the linear velocity that is the first component of the output vector *y*(*t*) from one of the four tested P3-DX robots. The top-left picture corresponds to a fixed sampling time of 10 ms, which shows good tracking performance. The top-right figure displays a high-performance self-triggered implementation (σ = 0.9). The bottom-left illustration depicts a low-performance self-triggered implementation (σ = 0.05). The bottom-right picture describes the adaptive self-triggered solution. It can be appreciated that the higher the value of cr, the better the servo control performance. Nevertheless, the adaptive self-triggering solution presents a balanced solution with a lower number of channel access and an acceptable control performance. The same behavior is observed for the tracking of the angular velocity that is the second component of the output vector *y*(*t*), which for the sake of space, is omitted from the paper.

Table 2 shows that the minimum transmission time for a channel with a high delay level is used to calculate the maximum transmission rate (*λ_max_* = *T_min_*^−1^) of each implementation. It can be appreciated that the adaptive solution offers a transmission rate similar to the low-performance self-triggered implementation.

A statistical study has been carried out to better characterize the validation procedure. The study consists of 350 simulations of each implementation. A fixed combination of linear and angular velocities values has been chosen as a reference while randomly switching the application time of each value. Table 3 summarizes the average and the standard deviation of performance and updates of the four robotic units, except for the case of periodic sampling that is only applied to one robot to be used as a reference for the rest of implementations. Comparing the previously-mentioned experiments, the results confirm the benefits of the authors' proposal. The improved aperiodic solution based on adaptive self-triggering provides an average number of updates clearly lower than the periodic case and nearly half of the self-triggered designed for high performance (σ = 0.9). However, the average ISE values are similar to those obtained with robots implementing the mentioned self-triggered approach and slightly worse than the periodic case.

### 5.2. Experimental Results

Experiments with four real P3-DX robots working on an IEEE 802.11g standard wireless network have also been carried out. The combination of linear and angular velocities of the previous sub-section is used. The experimental results were obtained only with the adaptive STC on the four P3-DX robots, due to the poor performance of the low sigma value (σ = 0.05) and the large transmission rate required for the high value (σ = 0.9), as well as the periodic sampling.

The WiFi network consists of an access point (AP) implemented by a router Buffalo WHR-HP-54 in the remote center. Moreover, each robot is provided by an Ethernet converter Buffalo WLI-TX4-G54HP working over the IEEE 802.11g. The transmission rate is set to auto-ranging from 1 to 54 Mbps. The router automatically uses the fastest possible data rate. The best possible connection speed is negotiated between the router and a wireless client. The CTS (clear to send)/RTS (request to send) protection mode is set up. When multiple devices are connected to an access point, they can occasionally be transmitting data to the access point at the same time, because no device can determine whether the other client is transmitting or not. When this happens, the AP discards both pieces of colliding data. The CTS/RTS protection mode avoids this issue by delegating which device gets to transmit at a given time. The beacon interval is set to 50 ms. A beacon packet is a packet broadcast by the router to synchronize the wireless network.

The main constraints of the channel are: packet dropouts due to packet collisions; other tasks running on the robot, introducing additional delays; other 802.11g networks generating interferences in the experimental area; *etc.*

A packet is considered lost when the delay is higher than the maximum delay (*τ_MAX_* =150 ms) or when the packet fails to reach its target. When a packet is lost, the robot sends a new one. With the adaptive self-triggered implementation; in the worst case scenario, the registered packet dropout is lower than 1%. This value does not threaten the stability of the system under study. Nonetheless, this problem will be addressed in detail in future works.

**Figure 8:**
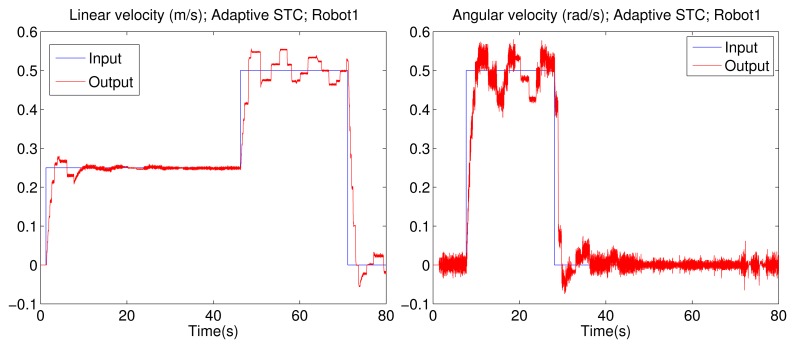
Linear and angular velocity registered (red line) when a reference (blue line) is applied to one of the four robots (simultaneously controlled by the remote center) with the adaptive solution proposed by the authors.

**Figure 9:**
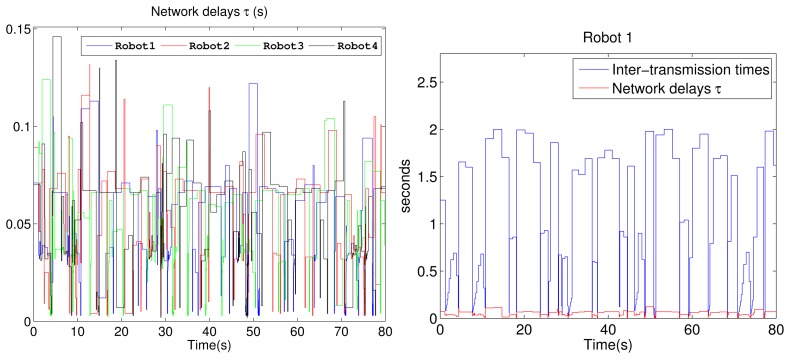
Actual network delays τ registered from the experimental test with four remotely-controlled robots (**Left**) and a detailed representation of the network delays τ and inter-execution times of Robot 1 (**Right**).

The linear and angular velocities from one of the P3-DX units with the adaptive aperiodic implementation are shown in Figure 8. It can be appreciated that the adaptive self-triggering solution presents an acceptable control performance quantified by the ISE value related to each robot control. The tracking error and the number of transmissions through the network are quantified in the “adaptive STC” of Table 4. Figure 9 shows the current network delays and the inter-execution times τ obtained from the experiment with four robotic units. The picture on the left confirms the erratic behavior of delays in a WiFi channel. The picture on the right shows the inter-execution times and the network delays for Robot 1. As can be appreciated, the higher the network delay, the higher the inter-transmission times and vice versa. This way, the proposed controller actively and dynamically contributes to optimizing the channel availability for control or other shared applications among several nodes.

Comparing the results obtained in the simulation (Figure 7, bottom-right) *versus* the experimental ones (Figure 8, left), it can be realized that those obtained with the P3-DX are slightly deteriorated in the performance of the control system due to the fact that the real wireless channel presents actual constraints not implemented in TrueTime. Consequently, the real wireless channel presents delays larger than the simulated ones, leading to conservative σ values in order to relieve the channel load (Table 1). This explains why the average number of updates is lower in the experimental scenario.

Table 4 also highlights the benefits of the authors' proposal with respect to the conservative approach [27], where only the maximum channel delay is considered to tackle with a self-triggered control solution. The performance requirements of the conservative approaches are highly restrictive, mainly when the maximum delay is large. This is because the *t_min_* obtained through the σ value has to be greater than the maximum channel delay (*τ_MAX_*) to guarantee the stability. To replicate the controller designed in [27], we took into account that the *τ_MAX_* with four robotics units was 150 ms; this is why we worked with σ = 0.1 (*t_min_* = 154.5 ms) when |*x*(*t*) − *x_eq_*| > 0.1 and σ = 0.05 (t*_min_* = 164.2 ms) when |*x*(*t*) − *x_eq_*| ≤ 0.1.

The mean values of communication updates and performance index obtained from Table 4 are compared with the periodic case (simulated results, sampling time equal to 10 ms); see Table 5.

Considering the same velocities references, with respect to the update number of the periodic case, the mean value of the new proposal is 97.5% lower; meanwhile, it is reduced 98.4% in the case of the conservative proposal [27]. Taking the ISE value of the periodic case as a reference, the tracking error of the new control solution is 24% higher; however, it is increased 806% with the conservative one.

## 6. Conclusions

Self-triggered control with compensation of channel delays based on its upper limit has solved different problems in the context of wireless CPS. This paper proves and illustrates that a non-conservative aperiodic alternative improves NCS applications when the channel delay is highly variable in cases, such as a WiFi networks. The key to this alternative is the double adaptation of the triggering mechanism considering the measured delay in each control iteration and how far from the steady state the system is. The main idea is to relax the triggering mechanism when the channel occupancy level increases and the state of the system under control is close to the equilibrium condition. In this way, the described strategy actively and dynamically contributes to optimizing the channel availability for control or other shared applications among several network nodes.

The benefits of this adaptive self-triggered solution have been tested in a multi-robot application using a unique remote controller, sharing a WiFi network. Firstly, the algorithm has been validated by simulation using the TrueTime tool and then by implementation on four P3-DX robotic units. The experimental results are slightly worse than the simulated ones due to the fact that the real wireless channel presents actual constraints not implemented in TrueTime. However, they enable the authors to demonstrate the advantages of their solution with real communication constrains. Additionally, the described solution allows the designer to estimate the maximum number of robotic units remotely controlled using the wireless network supported by the IEEE 802.11g standard. Obviously, a better adaptation of the triggering mechanism to the current channel delay requires splitting the gamma cumulative distribution function into more than three parts. In non-packet-based networks, the higher the number of *L* zones, the higher the size of information sent by the remote center to the robots. Summing up, the designer should be aware of this drawback regarding the increase of the data amount when the number of *L* zones is selected.

Currently, the authors are working on the application of this control strategy to non-linear systems, such as the trajectory tracking of robots' formation. Future work will involve the analysis of other channel communication problems, *i.e.*, packet dropout and the minimum stabilizing bit rate, on the stability of NCS.

## Figures and Tables

**Table 1:** Proposal of σ values for the adaptive self-triggered strategy.

		***CurrentDelay*** τ		

		**Zone 3**	**Zone 2**	**Zone 1**
|*x*(*t*) − *x_eq_*|	≤ 0.1	σ_1_ = 0.05	σ_2_ = 0.2	σ_3_ = 0.3
	> 0.1	σ_4_ = 0.1	σ_5_ = 0.7	σ_6_ = 0.9

**Table 2:** Maximum transmission rate with the three different implementations of the controller. STC, self-triggered control.

	**Periodic**	**STC σ = 0.05**	**STC σ = 0.9**	**Adaptive STC**
*T_min_* (ms)	10	164.2	14.3	154.5
λ*_max_* (Tx/s)	100	6.09	69.93	6.47

**Table 3:** Key parameters of four P3-DX robotic units for comparison of the average (AVG) and standard deviation (STD) values of 350 simulation results concerning the different control strategies.

	**Periodic STC σ = 0.05**			**STC σ = 0.9**		**Adaptive STC**	
AVG Updates	8000	100.85	100.91	699.36	695.21	266.36	311.38
		101.07	100.81	697.74	699.21	337.47	310.72

STD Updates	0	14.240	14.378	201.014	200.859	136.810	149.077
		14.255	14.423	203.268	203.375	160.829	150.305

AVG ISE	0.5633	12.2230	12.0764	0.6244	0.6259	0.6943	0.6047
		12.1246	12.1745	0.6246	0.6369	0.5936	0.6072

STD ISE	0.1070	6.8767	5.9323	0.1820	0.1717	0.2943	0.1214
		5.7710	6.2066	0.1607	0.2121	0.1126	0.1375

**Table 4:** Key parameter comparison of the experimental results obtained by the new proposal and by that described in [27]. ISE, integral of the squared error.

	**Authors' Proposal**		[27]	
Updates (WiFi Tx)	216	143	126	131
	264	167	119	132

ISE	0.7212	0.6893	5.3715	5.2637
	0.8066	0.6916	5.2490	5.2694

**Table 5:** Comparison of experimental results obtained applying the new proposal and that described by [27] with the simulated results obtained from periodic implementation.

	**Periodic (Simulated)**	**Authors' Proposal (Experimental)**	**[27] (Experimental)**
Updates (WiFi Tx)	8000	194.5	127
ISE	0.5819	0.7272	5.2694

## Supplementary Materials

Supplementary File 1. It can be accessed at: http://www.mdpi.com/1424-8220/15/6/12454/s1.
